# Genome editing through large insertion leads to the skipping of targeted exon

**DOI:** 10.1186/s12864-015-2284-8

**Published:** 2015-12-21

**Authors:** Borhan Uddin, Nan-Peng Chen, Marko Panic, Elmar Schiebel

**Affiliations:** Zentrum für Molekulare Biologie der Universität Heidelberg (ZMBH), DKFZ-ZMBH Allianz, Im Neuenheimer Feld 282, 69120 Heidelberg, Germany; The Hartmut Hoffmann-Berling International Graduate School of Molecular and Cellular Biology (HBIGS), University of Heidelberg, Heidelberg, 69120 Germany

**Keywords:** Genome engineering, Zinc-finger nucleases, CRISPR-Cas9, Exon-skipping, hCDC14A, hCDC14B

## Abstract

**Background:**

Highly efficient genome editing can be achieved through targeting an endonuclease to specific locus of interest. Engineered zinc-finger nuclease (ZFN) and CRISPR-associated protein-9 nuclease (Cas9) offer such an elegant approach for genome editing in vertebrate cells. In this study, we have utilized ZFN and Cas9-catalyzed double strand break followed by homologous recombination-mediated incorporation of premature stop codon and selection marker to target human cell division cycle 14A (*hCDC14A*) and cell division cycle 14B (*hCDC14B*) genes.

**Results:**

Targeting of the *hCDC14A* and *hCDC14B* loci in telomerase immortalized human retinal pigment epithelium (hTERT-RPE1) and human colon cancer (HCT116) cells were confirmed by Southern blot hybridization. Nevertheless, DNA sequence analysis of reverse transcription polymerase chain reaction (RT-PCR) products confirmed that in all the single/double allele ablations, the targeted exon was spliced out. The phenomenon of exon skipping was independent of the genome editing approaches exploited, Cas9 or ZFN. Because the exons had a nucleotide number that could be divided by 3, the reading frame of the exon deletion was maintained. This indicates an exon-skipping event possibly due to the insertion of large DNA fragment (1.7 to 2.5 Kb) within the targeted exons. As a proof-of-principle, we have used gene disruption followed by non-homologous end joining (NHEJ) approach. Small alterations in the exon (one to fifteen bases) were transcribed to mRNA without exon skipping. Furthermore, loxP site-mediated removal of selection markers left a 45 bp scar within the targeted exon that can be traced in mRNA without exon skipping.

**Conclusion:**

From this study, we conclude that insertion of a large DNA fragment into an exon by genome editing can lead to its skipping from the final transcript. Hence, more cautious approach needs to be taken while designing target sites in such that the possible skipping of targeted exon causes a frame-shift mediated incorporation of pre-mature stop codon. On the other hand, exon skipping may be a useful strategy for the introduction of protein deletions.

**Electronic supplementary material:**

The online version of this article (doi:10.1186/s12864-015-2284-8) contains supplementary material, which is available to authorized users.

## Background

Highly dynamic, yet tightly controlled, protein phosphorylation and de-phosphorylation events are important avenues to tweak cell cycle transition processes. Cyclin-dependent kinases (CDKs) lie at the heart of cell cycle control system and their activities rise and fall as the cell progresses through the cell cycle (Reviewed in [1]). In budding yeast *Saccharomyces cerevisiae*, the highly conserved ScCdc14 (cell division cycle 14 gene) phosphatase antagonizes the Cdk1 functions to allow anaphase regulation and mitotic exit [2]. Human cells encode three paralogs of *hCDC14* namely *hCDC14A*, *hCDC14B* and *hCDC14C* [3, 4]. In spite of the high conservations between the catalytic domain of all CDC14 phosphatases [5] and the complementation of *ScCdc14* by *hCDC14B* [6], human CDC14s have so far been reported to be involved in functions that are quite diverse than that of budding yeast [7]. Human hCDC14A was proposed to exert its function at centrosome duplication [8] while hCDC14B was implicated in mitotic progression [9], DNA damage checkpoint activation and DNA repair [10]. Nevertheless, hCDC14B depleted human cells display normal mitotic exit and cytokinesis [11]. Moreover, the viability of *hCDC14A* or *hCDC14B* single knockout (KO) vertebrate cells [12] indicate the possible functional redundancy of vertebrate phosphatases. It is noteworthy that most of the previously reported functions of hCDC14A/B were deduced upon siRNA depletion (often without a rescue experiment) or strong over-expression that causes toxic effects. Extent of depletion as well as the functional redundancy of the phosphatases was not taken into consideration partly because of the inability of available antibodies to recognize endogenous hCDC14A and hCDC14B proteins [12–14].

Genome editing provides an alternative strategy to siRNA depletion for hCDC14A and hCDC14B inactivation. Currently, several strategies exploiting sequence-specific endonucleases exist for purposeful genome editing, including Zinc-finger nuclease (ZFN), transcription activator-like effector nuclease (TALEN), and the RNA-guided clustered regularly interspaced short palindromic repeats (CRISPR)-Cas9 nuclease system [15]. In all these approaches, an endonuclease is programmed to specifically bind the assigned nucleotide sequence and trigger single or double strand breaks (DSB). Cells can take either high-fidelity homologous recombination (HR) and/or error-prone non-homologous end-joining (NHEJ) to repair this DSB [16] (Additional file 1: Figure S1). NHEJ might alter the DSB site by random insertion or deletion of nucleotides of varying length. On the other hand, homologous recombination can be utilized to deliberately introduce stop codons and selectable markers for ensuring genome disruption (Additional file 1: Figure S1).

In this study, we have used DSB-enhanced HR event to incorporate pre-mature stop codons followed by selection markers (~2 kb sizes) within the selected exons of human *hCDC14A* and *hCDC14B* genes. Nonetheless, Cas9 and ZFN-mediated genome disruption strategies have unfolded complications owing to the in-frame skipping of targeted exons. We presume that interruption due to the incorporation of a large fragment of DNA (1.7 to 2.5 kb including stop codon and selection markers) has structural effects on exon definition as reviewed by Valentine [17]. LoxP site-mediated removal of selection markers from the previously generated knockins prevented exon skipping but left a 45 bp Stop-loxP scar within the targeted exon. Thus, we report that insertion of large DNA fragment into an exon by genome editing leads to its skipping from the final transcript and how this property can be used in genome editing.

## Results and discussion

### Disruption of the *hCDC14A* and *hCDC14B* loci in human somatic cells

**Fig. 1:**
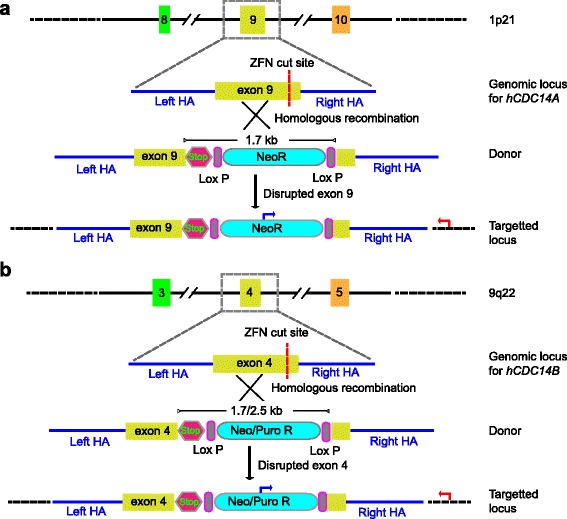
Strategy for ZFN-mediated generation of knockout cell lines. **a** Exon 9 of *hCDC14A* was targeted by zinc finger nuclease (ZFN). A donor template containing two homologous arms (HA), stop codon and neomycin selection cassette was used for their homologous recombination mediated insertion within the double strand break (DSB) site. Junction PCR with forward primer (blue arrow) in NeoR cassette and reverse primer (red arrow) in the genome outside homology arm confirmed successful targeting and insertion of the selection marker. **b** Exon 4 was targeted to knockout *hCDC14B* gene. As donor template for *hCDC14A,* neomycin selection cassette was used to generate *hCDC14B* single knockout. Puromycin cassette was used when *hCDC14B* was knocked-out on top of *hCDC14A* ^−/−^ cells (neomycin). Forward (blue) and reverse (red) primers used for the junction PCR are shown in the figure

**Fig. 2:**
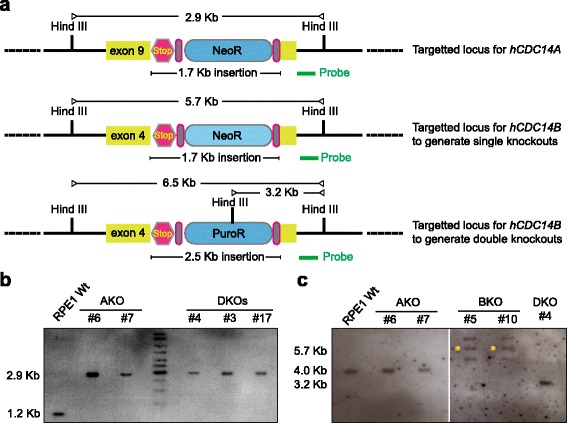
Southern blot hybridization to confirm biallelic targeting of *hCDC14A* and *hCDC14B* loci in RPE1 cells. **a** Map for *hCDC14A* and *hCDC14B* genomic locus targeted by zinc finger nuclease (ZFN). The probes for Southern blot hybridization were designed in the right homologous arms (shown as green bar). In case of *hCDC14A*, digestion by Hind III would result in a 2.9 kb fragment for knockout cells instead of wild-type 1.2 kb band. For knocking out *hCDC14B*, neomycin or puromycin inserted donor templates were used. Puromycin construct (with an extra Hind III site) was used when *hCDC14B* knocking out was carried out on top of *hCDC14A* ^−/−^ cells (NeoR) (double knockout). On the other hand, neomycin construct was used during generation of single *hCDC14B* knockout. **b** Southern blot hybridization to confirm *hCDC14A* knockout (AKO) with a *hCDC14A* specific probe (a). The expected *hCDC14A* band sizes were observed for wild type (Wt, 1.2 kb), *hCDC14A* single (AKO) and *hCDC14A hCDC14B* double knockouts (DKO) (2.9 kb). **c** Southern blot hybridization to confirm *hCDC14B* knockout (BKO) with a *hCDC14B* specific probe (a). The DKO (see a, PuroR) and Wt cells have shown anticipated band sizes of 3.2 kb and 4.0 kb, respectively. For single *hCDC14B* knockouts (BKO), two extra bands above and below the expected band size of 5.7 kb (asterisks) was persistently observed in different clones. Absence of wild type bands (4.0 kb) in these knockouts confirmed the successful targeting of both alleles

We have utilized CompoZr^TM^ ZFNs, designed and evaluated by Sigma Advanced Genetic Engineering Labs, to make *hCDC14A* and *hCDC14B* knockouts in two human cell lines (hTERT-RPE1 and HCT116) with stable genotype. ZFN-induced homologous recombination strategy was employed to incorporate premature stop codon and selection markers (neomycin or puromycin) into the targeted exons of *hCDC14A* (9^th^ exon) and *hCDC14B* (4^th^ exon) (Fig. 1a, b). Southern blot analysis has confirmed the successful biallelic targeting of the *hCDC14A* and *hCDC14B* loci by NeoR or PuroR cassettes (Fig. 2a-c).

### RT-PCR analysis confirming expression of wild type and in-frame exon-skipped *hCDC14A/ hCDC14B* transcripts

**Fig. 3:**
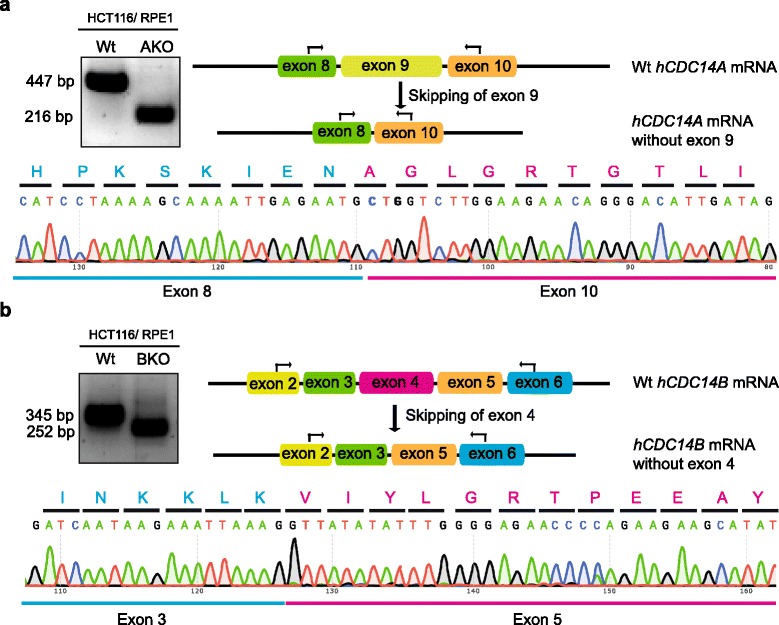
RT-PCR analysis to confirm expression of wild type and exon-skipped transcripts. Gel images for RT-PCR products of *hCDC14A* (**a**) and *hCDC14B* (**b**) transcripts from wild type (Wt) and knockout cells (KO). Maps show the locations of primers (black arrows) used for PCR reaction. The generated DNA bands were gel purified and sequenced. Sequences of alternating exon junctions are shown in the respective lower panels (exon 8 to 10 in case of *hCDC14A* and exon 3 to 5 for *hCDC14B*). In-frame exon skipping can be deduced from the amino acid sequences written above the codons

Sequencing of the *hCDC14A* and *hCDC14B* RT-PCR (reverse transcriptase polymerase chain reaction) products from wild type and selection marker knockins verified the presence of exon-skipped mature mRNA in the targeted hCDC14A and hCDC14B clones (Fig. 3a, b). The reading frames remain intact as the skipped exons in both the cases (9^th^ exon for hCDC14A and 4^th^ exon for hCDC14B) contained nucleotide number that could be divided by three. Hence, we have to assume the presence of truncated hCDC14A and hCDC14B proteins.

The 9^th^ exon of hCDC14A includes the active site cysteine and aspartate residues necessary for phosphatase activity [5, 18, 19], suggesting the inactivation of hCDC14A phosphatase from these cell lines. Conversely, the catalytically important residues for hCDC14B are located in 9^th^ exon, downstream to the targeted exon [5]. Thus, in case of hCDC14B the gene interference strategy most likely creates a small truncation of 31 amino acids in the hCDC14B protein that might not affect phosphatase activity.

### Exon-skipping phenomenon is independent of genome editing approach

**Fig. 4:**
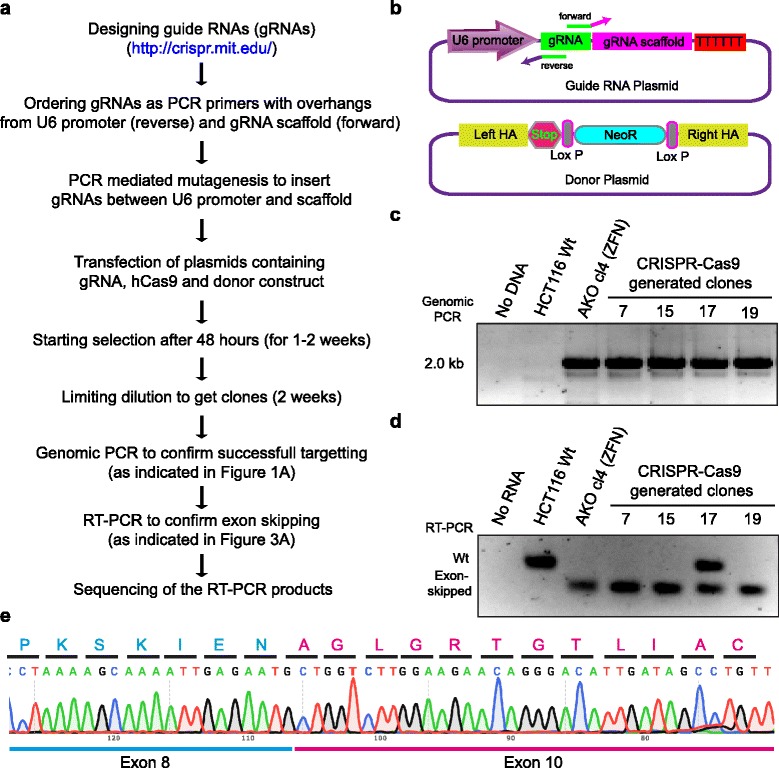
Strategy for Cas9-mediated generation of *hCDC14A* knockout HCT116 cells. **a**, **b** Workflow for CRISPR-Cas9 medicated insertion of NeoR into exon 9 of *hCDC14A*. Guide RNAs (gRNAs) targeting the exon 9 of *hCDC14A* gene were designed using the web tool (http://crispr.mit.edu/ [25]). ‘Churh gRNA insert’ containing the U6 promoter and gRNA scaffold [26] was first synthesized as gBlock and cloned into pJet. The intended gRNAs were inserted through PCR mutagenesis using primers indicated by arrows (top of **b**). The same donor construct (bottom of **b**) as in case of ZFN-mediated genome editing was used to target the locus. **c** Junction PCR (as in Fig. 1a) with forward primer in NeoR cassette and reverse primer in the genome outside homology arms confirmed successful targeting and insertion of the selection marker.**d** RT-PCR of purified mRNA from Wt and different CRISPR-Cas9 targeted *hCDC14A*-KO clonal cells. Primers were as in Fig. 3a. Presence of both wild type and exon-skipped RNA indicated the targeting of single allele in clone 17. The other NeoR clones 7, 15 and 19 contained bi-allelic knockout of the *hCDC14A* gene. **e** The generated DNA bands of the RT-PCR were gel purified and sequenced. Sequences of alternating exon junctions of *hCDC14A* are shown and in-frame exon skipping can be deduced from the amino acid sequences written above the codons

Our ZFN-mediated genome editing of *hCDC14A* and *hCDC14B* has clearly indicated the skipping of targeted exon from the final transcript. As a faster and more affordable alternative to ZFN, there is a recent surge in use of CRISPR-Cas9 system for genome editing. We have taken the advantage of CRISPR-Cas9 system to target *hCDC14A* locus with the same donor construct and guide RNA that targets the same genomic *hCDC14A* DNA sequence as the ZFN. As the lengthy custom design of the ZFN was avoided, CRISPR-Cas9 strategy was clearly faster and more than 50-fold cost-efficient than the ZFN approach. As anticipated, we observed exon skipping in HCT116 and RPE1 cells in which *hCDC14A* was targeted by CRISPR-Cas9 (Fig. 4, Additional file 2: Figure S2). This further implies that the skipping event is associated with the degree of exon alteration not merely a random outcome of genome editing approach.

### Minimizing the degree of alteration salvages the exon skipping

**Fig. 5:**
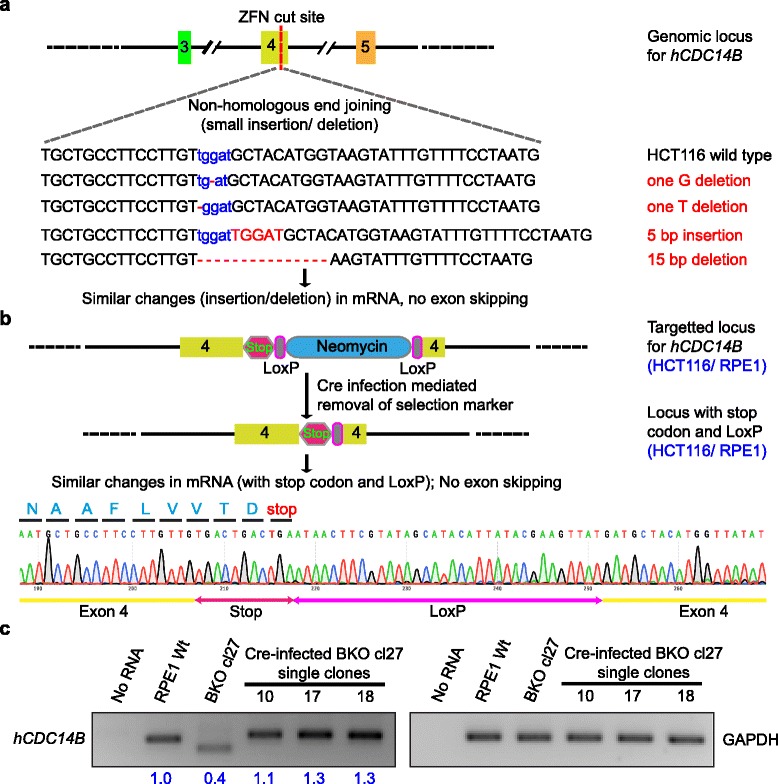
Exon skipping can be abolished by minimizing the degree of alteration. **a** Exon 4 of *hCDC14B* was targeted by ZFN to introduce double strand break (DSB) and error-prone NHEJ for random insertion and deletion. In some of the targeted loci, small alterations in the exon (one to fifteen bases) were observed. ZFN cutting sites are written in blue font and base deletions or insertions are marked by red font color. Sequencing of the RT-PCR products confirmed identical base pair changes without exon skipping (sequence not shown). **b** The selection cassette flanked by loxP sites was removed by Cre-recombinase from HCT116 and RPE1 *hCDC14B*-KO cells. RT-PCR analysis confirmed the presence of stop codon (11 bp) followed by one loxP site (34 bp) within the targeted exon in both the cell lines. **c** Semi-quantitative RT-PCR indicated that the exon-skipped mRNA level of RPE1 cells is less than half of the wild-type mRNA. 40 ng of total RNA was used and the input RNA level was confirmed by GAPDH amplification as mentioned by Carbery et al. [30]. The experiment was performed three times with similar outcome. One representative experiment is shown. The numbers below the agarose gel summarizes the relative abundance of the mRNA from three different experiments

As a cause of exon skipping, we hypothesized that large insertion-mediated alteration of the targeted exon affects the pre-mRNA structure [17]. Hence, to avoid large-scale genome editing, we have taken two experimental strategies. First, genomic loci were targeted by the same ZFNs but with error-prone NHEJ, with the view in mind to have random smaller insertions or deletions (Fig. 5a). Secondly, the selection markers flanked by loxP sites were removed by Cre-recombinase to generate loci with 45 base pair insertions including stop codons immediately followed by the single loxP site (Fig. 5b). In both the cases, sequencing of genomic loci indicated the expected abruptions. Similarly, sequencing of the RT-PCR products confirmed the likewise modifications within the targeted exons without their skipping from the mature transcript (Fig. 5a, b).

Semi-quantitative RT-PCR using GAPDH as a control indicated that the exon-skipped *hCDC14B* mRNA level was less than half of the wild-type mRNA (Fig. 5c). Such decrease in mRNA level might be due to transcriptional control (altered synthesis) and/or post-transcriptional regulation (altered stability) of disrupted cellular transcripts. Because of the lack of anti-hCDC14B antibodies that detect the endogenous proteins [12–14], we were unable to confirm this mRNA decrease at the protein levels.

Taken together, we describe that insertion of a relatively large DNA fragment into an exon can cause exon skipping. If the exon has a length that can be divided by 3, this leads to an in-frame fusion between the alternating exons. In case of hCDC14A, the truncated gene product lacks phosphatase activity because the deleted exon 9 contains the active site cysteine residue. Since phosphatases can have a scaffold function [20], analysis of this hCDC14A-Cys mutant can be used to uncover such a role. In contrast, deletion of the exon 4 of hCDC14B most likely results in a small truncation in the N-terminus of hCDC14B away from the nucleolar targeting sequence [7]. Since the residues required for hCDC14B activity were not affected, it is likely that this truncation does not affect the activity of the phosphatase. However, removal of the selection marker by Cre-induced recombination prevented exon skipping and disrupted gene function by introducing stop codons. Thus, adenovirus-Cre mediated infection of hCDC14B::neomycin cells can be used to introduce an acute knockout of hCDC14B function.

## Conclusion

Genome editing is a robust experimental paradigm to assign cellular function(s) to a molecule. Simplicity of genome editing through engineered nucleases has recently allured the scientific community to generate knockout cellular models. Utilizing selection markers greatly augments the ease of generating knockout cellular clones. However, our study clearly shows that, the selection marker also imposes an inherent size-associated mRNA processing difficulty of the targeted exon. In most of the cases, the selection markers overweigh the average length of eukaryotic exon (<200 bp, [21, 22]). For example, the 4^th^ exon of *hCDC14B* is 93 bases long compared to the 1.7 kb of neomycin and 2.5 kb of puromycin cassette. Such massive alterations have the potential to annihilate secondary pre-mRNA structure and impede cellular mRNA processing leading to exon skipping. Well-informed and pre-planned experimental choices should be made to target the suitable exon while editing the genome of higher eukaryotes like in human cells. An alternative strategy could be the introduction of double strand break in an intronic sequence to incorporate homologous recombination mediated subtle changes in the adjacent exon. These exon modifications could be introduced in the donor along with the selection marker. Of note, such skipping mechanisms can be useful to engineer proteins with small deletions or for the analysis of acute knockouts in response to Cre - induced selection marker removal.

## Methods

### Generation of ZFN-mediated knockout cells

Human *CDC14A* and *CDC14B* specific CompoZr™ knockout Zinc Finger Nucleases were designed and evaluated by Sigma Advanced Genetic Engineering Labs. The ZFN for *hCDC14A* (product Number: CKOZFND2170-1KT) targets the sequence 5′-AGCACACCCAGTGACaacatCGTGCGAAGGTTCCTGAA-3′ in the 9th exon (cutting site in lower cases). Target sequence for ZFN against *hCDC14B* is in the 4th exon 5′-TGCTGCCTTCCTTGTtggatGCTACATGGTAAGTATTTG-3′ (product Number: CKOZFND5769-1KT).

The donor vector was constructed by PCR amplification of the genomic locus as reported by Panic et al. [23]. In short, the genomic locus 800 bp upstream and downstream of the ZFN cut site was amplified and sub-cloned into pJet 1.2 vector. The insertion cassette including STOP codon in every frame and neomycin/ puromycin resistance was inserted at the cut site of donor vector.

10^6^ cells were co-transfected with the ZFN mRNAs (2.5 μg of each) and donor vector (7 μg) by electroporation (Invitrogen, Neon transfection system). After electroporation, cells were cultured for 24 h at 37 °C (recovery) followed by 48 h at 30 °C (enhancing ZFN efficiency [24]). Cells were further cultured at 37 °C for 72 h prior to single cell dilution (limiting) in 96 well plates (500 μg/ml neomycin). Two weeks later, the emerging clones were screened by genomic PCR and positive clones were further confirmed by RT-PCR as well as southern blot hybridization. Junction PCR with one primer in donor construct and the other in the genome outside homology arm confirmed successful targeting and insertion of the selection markers. The following primers were used for junction PCR - hCDC14A: forward 5′-CGGCTATGACTGGGCACAAC-3′; reverse 5′- GCCTCCTCGAAGTCAAACAAG −3′; hCDC14B (for NeoR insertion): forward 5′-CGGCTATGACTGGGCACAAC-3′; reverse 5′-CGATCTCCGCTCACTG-3′; hCDC14B (for PuroR insertion): forward 5′-CGGGGCGAAGGCAAC-3′; reverse 5′-CGATCTCCGCTCACTG-3′. Similar approach without the donor construct was taken in case of NHEJ-facilitated disruption of *hCDC14B* gene. The resultant insertion/deletion-mediated mutagenesis was detected by sequencing of the amplified (Q5® High-Fidelity DNA Polymerase - NEB) genomic locus surrounding the ZFN cut site (primers used - forward 5′-TGAATGGTTATGGGATTTGGA-3′; reverse 5′-GCACAGCTTCCTTGAATTGG-3′).

### Generation of Cas9-mediated knockout cells

Guide RNAs (gRNAs) targeting the exon 9 of *hCDC14A* was designed using the web tool (http://crispr.mit.edu/ [25]). gRNA1 (5′-CCAGTGACAACATCGTGCGA-3′), gRNA2 (5′-CCTTCGCACGATGTTGTCAC-3′) and gRNA5 (5′-CTTCGCACGATGTTGTCACT-3′) with scores 96, 91 and 86, respectively, were selected as they were exactly targeting the ZFN binding site (5′-AGCACACCCAGTGACaacatCGTGCGAAGGTTCCTGAA-3′). ‘Churh gRNA insert’ containing the U6 promoter and gRNA scaffold [26] was first synthesized as gBlock from IDT (http://www.idtdna.com/pages/products/genes/gblocks-gene-fragments) and cloned into pJet1.2 vector. The intended gRNAs were then ordered as PCR primers with overhangs from U6 promoter and gRNA-scaffold and inserted between U6 promoter and gRNA scaffold through PCR mutagenesis (Fig. 4).

Different strategies were used to generate *hCDC14A*-KO HCT116 and RPE1 cells. For HCT116, hCas9 plasmid (a gift from George Church (Addgene # 41815, [26])) was transiently transfected with gRNA and donor plasmids. Whereas for RPE1 cells, pCW-Cas9 plasmid (a gift from Eric Lander & David Sabatini (Addgene # 50661 [27])) containing doxycycline (Dox) inducible spCas9 was lentivirally integrated into RPE1 FRT/T-Rex cells. Successful expression and nuclear localization of Cas9 was confirmed by indirect immunofluorescence and western blot analysis (Additional file 2: Figure S2). The cells were then electroporated with the plasmids containing gRNA and donor vector as showed in Fig. 4b. Junction PCR with forward primer in NeoR cassette (5′-CGGCTATGACTGGGCACAAC-3′) and reverse primer in the genome outside homology arms (5′-GCCTCCTCGAAGTCAAACAAG-3′) confirmed successful targeting and insertion of the selection marker.

### Southern blot hybridization to confirm knockouts

Genome editing in both RPE1 and HCT116 cells was verified by Southern blot hybridization [28]. Genomic DNA was isolated using MasterPure DNA purification kit (Epicentre, Cat MCD85201) following manufactures instructions. 20 μg of DNA were digested overnight with FastDigest Hind III-HF (Thermo Scientific) and run overnight onto a long (18 cm) 0.8 % agarose gel at 30–35 V. The gel was subsequently stained and photographed (fluorescent ruler of gel-casting tray was used to track the distance of migration of DNA bands). The gel was then washed in double distilled water (ddH_2_O) and DNA was denatured in 0.5 M NaOH, 1.5 M NaCl (twice for 20 min with gentle shaking). After rinsing once with ddH_2_O, DNA was neutralized by washing thrice for 15 min with 1.5 M NaCl, 0.5 M Tris–HCl (pH 7.0) and transferred onto a GeneScreen Plus® Hybridization Transfer Membrane (PerkinElmer) by overnight capillary transfer in 10xSSC buffer (1.5 M NaCl, 0.15 M trisodium citrate). After rinsing the membrane with 2XSSC buffer, DNA was UV-crosslinked with a Stratalinker 1800 (Stratagene).

The membrane was hybridized over night in DIG Easy Hyb buffer (Roche, cat#11603558001) with DIG labeled probe generated by PCR DIG probe Synthesis kit (Roche, cat#11636090910). Primers used to generate DIG probes for *hCDC14A*: forward 5′-CATCGCCGTTCACTGC-3′, reverse 5′-ACGTGGGCCTGGAAAG-3′; and *hCDC14B*: forward 5′-GCCCAACTACTTTGGCAAAG-3′, reverse 5′- CCAATGATCCAAATGGAGCAC-3′.

### RNA preparation from cells and expression analysis

Total RNA was isolated from cells using the RNeasy Mini Kit (Qiagen, Hilden, Germany) following manufacturers protocol. RT-PCR analysis confirming expression of wild type and exon-skipped *hCDC14A/B* transcripts were carried out using SuperScript® III One-Step RT-PCR System with Platinum® Taq DNA Polymerase (Invitrogen). Forward primer binding to 8^th^ exon (5′-ATGGTGACTTCAACTGGA-3′) and reverse primer binding to 10^th^ exon (5′-CTTCCAGGAAGTGCTGC-3′) were used for RT-PCR of *hCDC14A* coding sequence (CDS). In case of *hCDC14B,* the forward primer was designed in the 2^nd^ exon (5′-GCCATTCTCTACAGCAG-3′) while reverse primer in 6^th^ exon (5′-GCAACTTCCATAGGCAGC-3′). Bands corresponding to wild type and exon-skipped clones were excised from the gel and sequenced to verify the presence of the splice junctions (*hCDC14A*: exon 8 → 10; *hCDC14B*: exon 3 → 5). Human *GAPDH* was amplified with the primers described by Zhang et al. [29]: forward 5′-ATCCCATCACCATCTTCCAG-3′ and reverse 5′-CCATCACGCCACAGTTTCC-3′. For semi-quantitative RT-PCR, 40 ng of total RNA was used as template and the relative amount of mRNA in different samples was calculated by measuring the band intensity (normalized to GAPDH) using NIH ImageJ software [30] .

### Immunofluorescence microscopy and immunoblotting

Cells were seeded on cover slips, allowed to attach and fixed with ice-cold methanol for 5 min at −20 °C prior to permeabilization with 0.1 % Triton X-100 for 10 min. After, blocking with 10 % (v/v) fetal calf serum (FCS) for 30 min, cells were stained with anti-bodies in 3 % (w/v) BSA (bovine serum albumin) in PBS. Hoechst 33342 (0.2 g/ ml, Calbiochem) was used to visualize nucleus. The anti-Flag (Cell Signalling, M2) primary antibody was used to detect 3XFLAG-SpCas9. Imaging was performed on a DeltaVision RT system (Applied Precision) with an Olympus IX71 microscope using Softworx software (Applied Precision).

For immunoblotting, cells were washed twice with PBS and lysed in lysis buffer (10 mM Tris-Cl pH 7.5, 150 mM NaCl, 5 mM EDTA, 0.1 % SDS, 1%Triton X-100, 1 % deoxycholate) supplemented with 1 mM PMSF (Sigma) and protease inhibitor cocktail (Roche) for 30 min. Lysates were centrifuged and the supernatant was boiled with Laemmli buffer. SDS-PAGE was performed as previously described [31]. The membranes were blocked in 5 % non-fat milk in TBS-T and anti- α-tubulin (Sigma, T-9026), anti-Flag (Cell Signalling, M2) primary antibodies were used with appropriate secondary antibodies.

### Cell culture

hTERT-RPE1 cells were cultured in Dulbecco’s Modified Eagle’s Medium (DMEM) F12 (Gibco) supplemented with heat inactivated 10 % fetal bovine serum (FBS), 100 U/ml penicillin and 100 mg/ml streptomycin. HCT116 cells were cultured in McCoy’s 5A (Gibco) medium supplemented with 10 % FBS, 100 U/ml penicillin and 100 mg/ml streptomycin. All cell lines were cultured at 37 °C with 5 % CO_2_.

### Ethics statement

No ethics approval was required for this study.

### Availability of supporting data

The supporting sequencing files for Figs. 3, 4 and 5 are attached as supplementary files with the manuscript.

## Additional files

Additional file 1:
**Figure S1.** Schematic illustration of genome editing by engineered nucleases. Engineered nucleases like zinc finger nucleases (ZFN), transcription activator-like effector nuclease (TALEN), and the RNA-guided clustered regularly interspaced short palindromic repeats (CRISPR)-Cas9 nuclease systems are targeted to specific nucleotide sequences to cause double strand break (DSB). Cells can repair this DSB either by high-fidelity homologous recombination (HR) and/or error-prone non-homologous end-joining (NHEJ) approaches [16]. Homologous recombination event can be manipulated to cause specific genome editing by introducing a donor template. On the other hand, NHEJ might disrupt gene function due to the random insertion or deletion of nucleotides of varying length (PDF 432 kb) Additional file 2:
**Figure S2.** Strategy for Cas9-mediated generation of *hCDC14A* knockout RPE1 cells. (A) Workflow for the construction of RPE1 *hCDC14A*-KO cell line. pCW-Cas9 plasmid containing doxycycline (Dox) inducible spCas9 was lentivirally integrated into RPE1 FRT/T-Rex cells. (B, C) Successful expression and nuclear localization of Cas9 was confirmed by indirect-immunofluorescence (B) and western blotting (C). (D) Junction PCR with forward primer in NeoR cassette and reverse primer in the genome outside homology arm (as in Fig. 1a) confirmed successful targeting and insertion of the selection marker. Exon skipping was confirmed by RT-PCR (primers as in Fig. 3a). Presence of both wild type and exon-skipped RNA indicated the targeting of single allele in clones 1 and 20. (PDF 1952 kb)

## Abbreviations

CRISPR: Clustered regularly interspaced short palindromic repeats
CDS: Coding sequence
Cas9: CRISPR-associated protein-9 nuclease
CDKs: Cyclin-dependent kinases
DSB: Double strand breaks
HA: Homologous arm
HR: Homologous recombination
hTERT-RPE1: Human telomerase reverse transcription–immortalized retinal pigment epithelial cells
hCDC14: Human cell division cycle 14
NHEJ: Non-homologous end joining
RT-PCR: Reverse transcription polymerase chain reaction
TALEN: Transcription activator-like effector nuclease
ZFN: Zinc-finger nuclease
