# Supplementary figures and images for: Genome editing through large insertion leads to the skipping of targeted exon
Source: BMC Genomics. 2015 Dec 21;16:1082. doi: 10.1186/s12864-015-2284-8 (PMC4687116; doi:10.1186/s12864-015-2284-8)

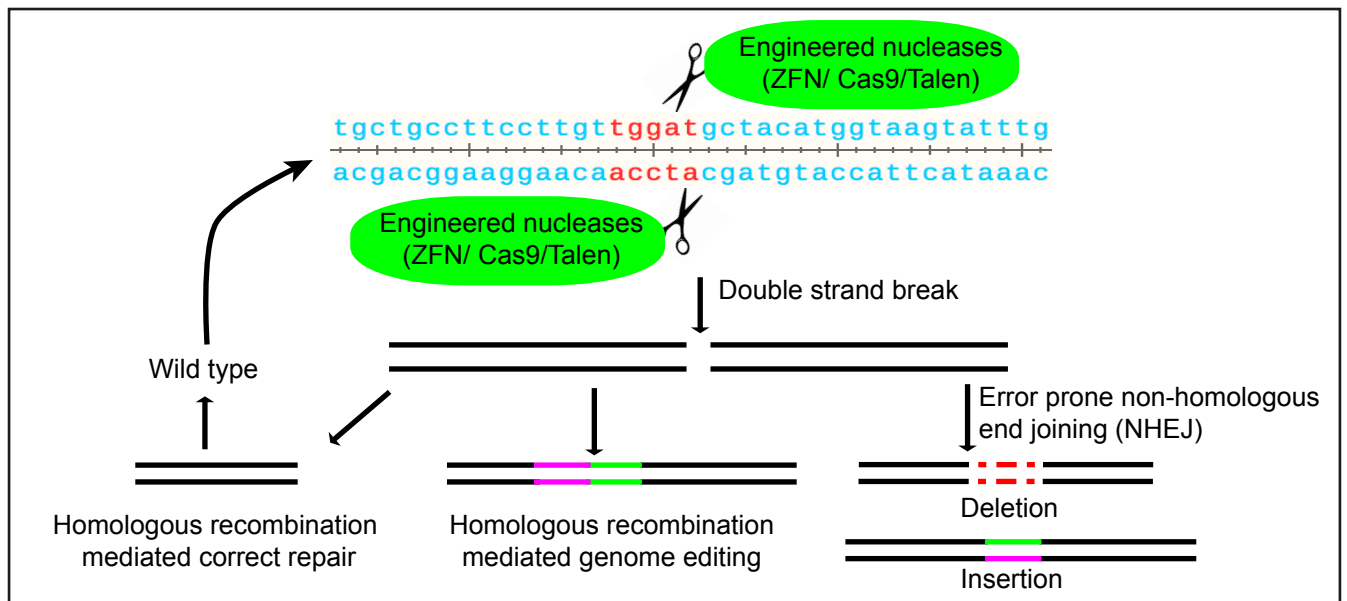

Supplement: Additional file 1: — Figure S1. Schematic illustration of genome editing by engineered nucleases. Engineered nucleases like zinc finger nucleases (ZFN), transcription activator-like effector nuclease (TALEN), and the RNA-guided clustered regularly interspaced short palindromic repeats (CRISPR)-Cas9 nuclease systems are targeted to specific nucleotide sequences to cause double strand break (DSB). Cells can repair this DSB either by high-fidelity homologous recombination (HR) and/or error-prone non-homologous end-joining (NHEJ) approaches [16]. Homologous recombination event can be manipulated to cause specific genome editing by introducing a donor template. On the other hand, NHEJ might disrupt gene function due to the random insertion or deletion of nucleotides of varying length (PDF 432 kb) [file 12864_2015_2284_MOESM1_ESM.pdf]

Suppl. Figure 2

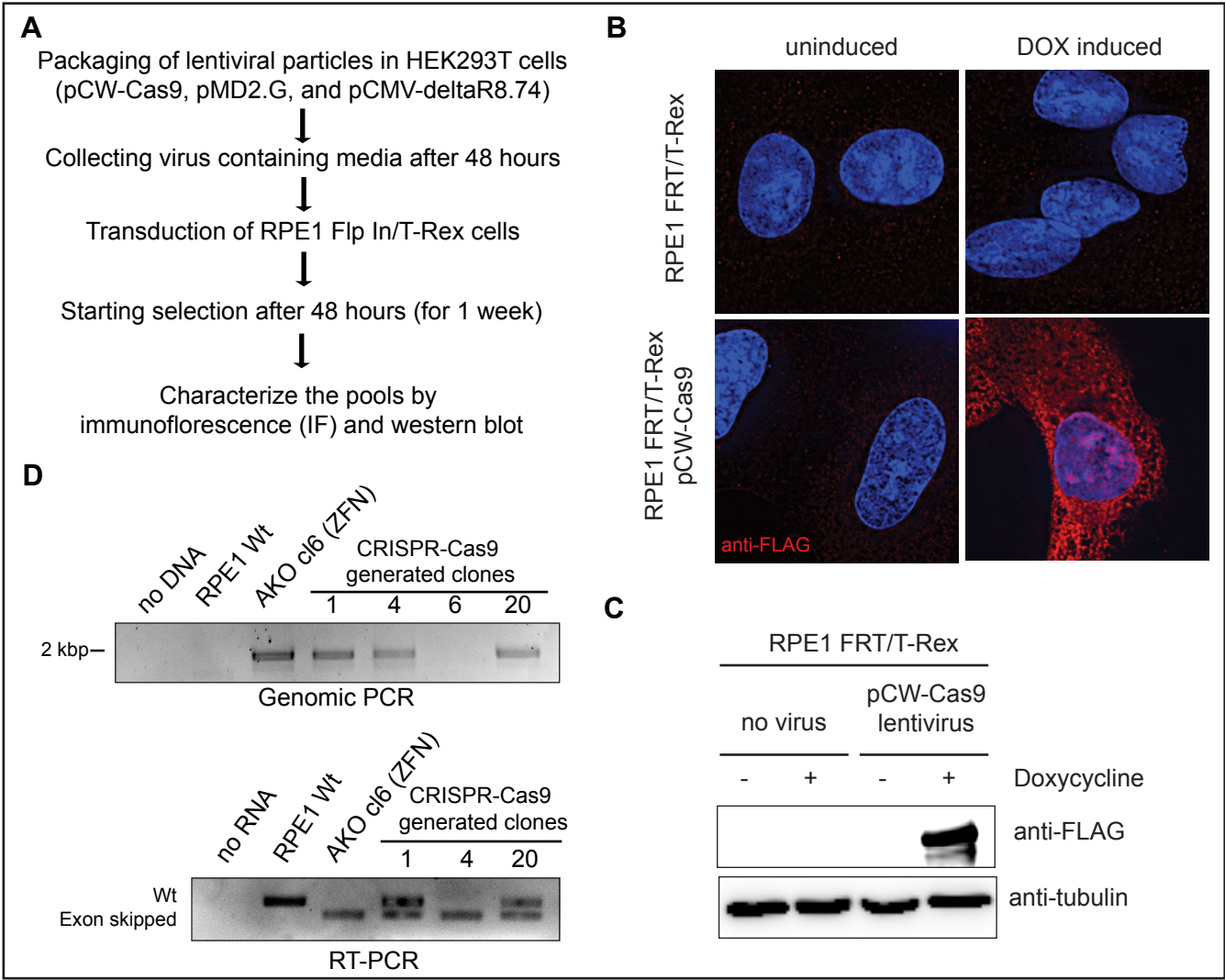

Supplement: Additional file 2: — Figure S2. Strategy for Cas9-mediated generation of hCDC14A knockout RPE1 cells. (A) Workflow for the construction of RPE1 hCDC14A-KO cell line. pCW-Cas9 plasmid containing doxycycline (Dox) inducible spCas9 was lentivirally integrated into RPE1 FRT/T-Rex cells. (B, C) Successful expression and nuclear localization of Cas9 was confirmed by indirect-immunofluorescence (B) and western blotting (C). (D) Junction PCR with forward primer in NeoR cassette and reverse primer in the genome outside homology arm (as in Fig. 1a) confirmed successful targeting and insertion of the selection marker. Exon skipping was confirmed by RT-PCR (primers as in Fig. 3a). Presence of both wild type and exon-skipped RNA indicated the targeting of single allele in clones 1 and 20. (PDF 1952 kb) [file 12864_2015_2284_MOESM2_ESM.pdf]
